# Self-reported health status in the general population over 2 decades: variation in EQ-5D-3L in Health Survey for England

**DOI:** 10.1186/s12889-025-24238-2

**Published:** 2025-09-30

**Authors:** Ling-Hsiang Chuang, Ranjeeta Thomas, Paul Kind

**Affiliations:** 1 Department of Epidemiology and Global Health, Umeå University, Umea, Sweden; 2 LHC Healthcare Consultancy, Utrecht, the Netherlands; 3 Department of Health Policy, London School of Economics, London, UK; 4 Department of Applied Health Research, University College London, London, UK; 5 Academic Unit of Health Economics, University of Leeds, Leeds, UK

**Keywords:** Population norm/Reference, EQ-5D-3L, Self-reported health status, Temporal variation, Health Survey for England

## Abstract

**Background:**

Population reference data based on self-reported EQ-5D constitute a valuable resource in a broad range of settings. Within the UK, EQ-5D has been included in national population surveys for over 30 years, notably as part of the Health Survey for England, however, the extent to which such data varies over time remains largely unknown.

**Methods:**

Between 1996 and 2014, the Health Survey for England included the original 3-level version of EQ-5D (EQ-5D-3L) and provides data from more than 100,000 respondents; these data form the basis of the present study. Age-stratified rates of reporting any problem in EQ-5D dimensions were computed for men and women; these were standardised using 1996 as the index year. Logit regression was used to examine the influence of year respondent socio-demographic characteristics, and current health status on rates of reporting any problems in EQ-5D-3L. The data of self-rated health status recorded on a 0-100 scale (EQ VAS) was also analyzed.

**Results:**

More than 30% report a problem with Pain/Discomfort with around 20% reporting a problem with Anxiety/Depression, Mobility having a similar problem rate, about 18%. Some 5% of respondents report a problem with Self Care. After an initial fall from 1996, self-reported health remains relatively stable across 10 years, however between 2008 and 2012 rates of reporting any problem increased, particularly in the Anxiety/Depression dimension and amongst older women. Logit regression analysis demonstrates that most of the covariates had statistically significant coefficients, such as age, gender, education, economic activity, income, and long-standing illness/condition.

**Conclusions:**

The study demonstrates the stability of EQ-5D responses over time in HSE data from 1996 to 2014. However, there is evidence of periodic deterioration in health status notably in the years immediately after 2007. Further investigation of this effect could have implications for the interpretation and use of population data based on EQ-5D. The study demonstrates the importance in national surveys of the general population of regular collection of health status data using a standardised measure of health-related quality of life.

**Supplementary Information:**

The online version contains supplementary material available at 10.1186/s12889-025-24238-2.

## Introduction

For population health the goal of preventing and treating disease is to improve function and reduce symptoms. Any change in the health of a population could be captured by periodic reassessment of health - for example, whether the population is achieving national targets for health [1]. Furthermore, such data, normative population reference data for health, play a key role in a wide range of settings, including the design of clinical studies and the analysis of health outcomes, as well as in a multitude of public health and policy functions, providing cross-national comparisons, demonstrating regional variations, and assessing the extent and nature of health inequalities.

EQ-5D [2] is a generic measure of health-related quality of life that has been widely used in population surveys following the Measurement and Valuation of Health (MVH) study conducted in 1993 [3] generated the first national reference data [1]. Over the following years several UK national surveys collected EQ-5D data on an occasional basis, including ONS Omnibus Surveys [4] and the General Household Survey [5]. From 1996 until 2014, EQ-5D was included in several editions of the Health Survey for England (HSE) [6]. These data provide an important cross-sectional representation of health over almost 2 decades.

Whilst in the short run it may be reasonable to assume that population health is unlikely to demonstrate significant variation, over longer periods of time when there are evident changes in social, environmental, and economic factors, some variation in self-reported health status might be expected. Population reference data for EQ-5D were initially based on the original MVH study as reported in 1998 [3, 7] and despite having been updated more recently [8, 9] the original paper continues to be widely cited. The question raised here is whether it remains reasonable to rely on data of such vintage and more generally to consider the extent to which self-reported health status reported in the general population varies over time.

Despite EQ-5D normative reference data having been published for several countries [10] as far as is known little is known about their variation over time; this partly stems from a general absence of sequential population studies, hence the importance of the interrupted time series created by pooling HSE data. Two specific questions can be addressed thereby; firstly, what degree of variation is observed in rates of self-reported problems; secondly, does the value of self-assessed health elicited by EQ-5D remain unchanged over time?

## Methods

### Study sample

Health Survey for England (HSE) [6] is an annual cross-sectional survey based on a random sample of private households in England. It excludes people who are unhoused or living in communal buildings such as nursing homes. HSE uses a standard sampling methodology [11] with participants being interviewed at home, with a nurse visit being arranged for those who agree to the collection of more detailed clinical information such as height, weight and blood pressure. HSE includes a set of core questions asked each year on a variety of health and health behaviors as well as demographic and socio-economic indicators,

A total of ten HSE surveys (1996, 2003–2006, 2008, 2010–2012, 2014) included EQ-5D-3L and these surveys provided the data for this study. Data were extracted on a range of socio-demographic characteristics, including age, gender, marital status, level of educational attainment, economic activity status, total household income, government office region and multiple deprivation index. Health-related information was also extracted including respondents’ weight, presence of long-standing illness, number of days with acute illnesses, medically diagnosed conditions and self-rated health status on a 5-point rating scale (very good, good, fair, bad, very bad). Amongst these 10 HSE surveys, where EQ-5D-3L was included, EQ VAS was only collected in the HSE survey for years 2010–2012 and 2014 and had been omitted from all other years.

### EQ-5D

EQ-5D is a generic measure of health-related quality of life defined by a descriptive classification of health based on 5 dimensions (Mobility, Self-Care, Usual Activities, Pain/Discomfort and Anxiety/Depression)^2^. In its original format (EQ-5D-3L) each dimension has 3 response levels (no problems, some problems, extreme problems); taken together, this classification system defines a total of 243 unique EQ-5D-3L health states. Problem levels on each dimension are combined to identify an individual respondent’s EQ-5D health state. Data are captured using a 2-part questionnaire in which respondents firstly indicate their level of problem on each dimension (EQ-5D self-classifier) and secondly, rate their own health on a vertical, 20 cm visual analogue scale calibrated with the endpoints ‘Best imaginable health state’ and ‘Worst imaginable health state’, scored 100 and 0 respectively (EQ VAS).

### Statistical analyses

Data from 10 HSE surveys were pooled and participants who provided complete responses to all 5 dimensions of EQ-5D-3L were identified. Descriptive analyses on socio-demographic and health-related variables and EQ-5D-3L problem response rates were conducted. Differences in socio-demographic and health-related variables between years were compared using chi-square tests, one-way ANOVA or Kruskal-Wallis test where appropriate. Age-stratified rates of reporting any problem in EQ-5D-3L dimensions were separately computed for men and women in each time period. To adjust for differences in age/gender in each wave, these distributions of each wave were adjusted to match those in the index year (1996). Logit regression was used to examine the impact of year and other socio-demographic/health characteristics on the variation of reporting any problems in EQ-5D-3L. Similar analyses were conducted for the EQ VAS, including age and gender stratified scores, means for most frequently occurring EQ-5D-3L health states (defined as more than 50 observations in one of year and gender stratified categories), and ordinary least square (OLS) regression. Analysis of EQ VAS was restricted to the years 2010–2012 and 2014 given its exclusion in all other years. EQ VAS norms based on the year 2014 were calculated by age and gender for proportion of responses in each dimension. For completeness of reporting, EQ-5D index scores are reported, based on the UK 3 L value set [13]. To examine variations in self-reported health status over time, respondent height was selected as a control variable on the assumption of its short-run stability [12].

Since 2003, sampling weights have been provided in the HSE dataset in order to deal with missing data and to enhance survey representativeness; as a sensitivity analysis, all aforementioned analyses were repeated with sampling weights.

## Results

A total of 100,825 records, with complete EQ-5D-3L responses were identified in the pooled HSE data. Top-level descriptive results of socio-demographic variables are presented in Table 1. It should be noted that in 2005 the survey focused on the health of older populations and over-sampled participants aged 65+, consequently the study sample has more widowed/retired individuals, with lower education and household income, more long-standing illnesses and morbidity than the other survey years. Over the study period, the mean age of the sampled population increases in successive years, but the gender balance remains more or less constant. Fewer respondents have only basic schooling and more have degree equivalent education; some two-thirds of respondents report being married or living with a partner. The overall composition of the achieved HSE samples appears to be broadly similar across the study period. Respondent height was selected as a control variable to provide an indication of consistency in sampling across the study period, working on the assumption that it should show minimal short-run variation. The mean heights for men and women were computed for all years and with the obvious exception of 2005, display an average invariance of less than 0.5 cm (see Supplementary Material Fig. 1. Average height over time).

**Table 1:** Primary characteristics of population surveys (% unless otherwise specified)

Year	1996	2003	2004	2005	2006	2008	2010	2011	2012	2014
*Sample size*	15,476	13,753	6,114	9,211	12,926	14,117	7,332	7,517	7,294	7,085
**Age** Mean age (yrs)	47.5	47.7	48.9	54.5	48.9	48.8	49.3	49.1	49.8	49.7
**Gender** % female	55.0	55.7	57.3	55.3	55.5	55.5	56.1	56.3	55.6	56.1
**Educational attainment**										
Degree equivalent	15.9	16.7	18.2	16.4	19.9	19.8	22.3	24.4	25.8	26.3
Basic schooling	37.2	25.6	28.4	33.4	25.9	25.1	20.8	21.4	21.2	20.5
**Economic status**										
In paid employment	53.0	57.2	55.3	43.5	55.6	55.4	54.1	55.5	53.5	55.2
Unemployed	3.8	4.8	4.2	3.1	4.2	4.5	5.1	4.8	5.4	3.9
**Marital status**										
Married/living together	67.5	64.1	64.3	63.7	65.4	64.2	65.6	64.0	63.7	64.0
Single	16.8	19.4	18.2	15.9	18.1	19.0	18.4	18.8	19.5	19.2
**Height (cm)**		167.3	167.0	166.5	167.5	167.5	167.6	167.6	167.6	167.7

Response levels for each dimension were dichotomized by collapsing levels #2 and #3 to create an “any problem” category. (Rates are presented in Supplementary Material Table 1). The pattern shows that Pain/Discomfort and Anxiety/Depression are the most frequently reported, with more than 30% and 20% respectively. Problem levels for Mobility and Anxiety/Depression are both about 18%. Around 5% of respondents report a problem with Self Care. The small proportion of respondents self-reporting as level #3 (“confined to bed”) on Mobility is consistent with a survey methodology based on face-to-face interviews, were potential respondents are unable to answer the door; this response category is poorly specified and may have limited practical value in a survey of the general population as only 117 (< 1%) of all respondents reported this level of restricted mobility. A similar observation attaches to level #3 in the Self-Care dimension (unable to wash/dress) where rates of around 0.5% are observed.

So as to compensate for variation in the age/gender composition of each HSE separate survey, respondents were grouped into 10-year age bands. Age/gender specific rates were then computed for each of 7 age-groups and are shown in Tables 2 and 3. The highest rates across years for each age group are highlighted and it can be seen that rates in 2011 are frequently greater than for other years. Amongst male respondents more than half (18/35) of the highest problem rates occur in 2011 with 5/7 age groups for Mobility and Anxiety/Depression recording highest across the HSE sample years. A similar phenomenon is evident for female respondents in 2011 with 60% (21/35) of age groups recording the highest rates., most obviously for Anxiety/Depression. Pain/Discomfort and Mobility demonstrate similar patterns.

**Table 2:** Age-stratified ANY problem rates (Men)

Men	1996	2003	2004	2005	2006	2008	2010	2011	2012	2014
***Mobility***										
16–24	5.6	2.6	**5.8**	3.8	2.9	3.2	3.9	4.5	3.7	5.0
25–34	5.2	5.9	3.1	4.8	3.4	4.3	5.4	4.5	5.6	**6.2**
35–44	7.7	8.6	6.5	8.7	8.0	9.4	6.9	**9.9**	7.6	9.5
45–54	12.3	12.1	14.9	13.7	13.9	11.9	13.8	**15.1**	13.0	11.9
55–64	26.6	23.3	20.1	23.5	22.5	21.1	25.4	**26.9**	23.2	20.2
65–74	32.0	29.8	30.0	29.9	31.0	32.0	28.4	**33.6**	25.8	25.3
75 +	46.4	47.9	47.4	42.4	48.4	48.0	43.8	**52.8**	39.5	46.0
***Self-care***										
16–24	**1.8**	0.9	0.4	0.3	1.0	0.56	0.6	1.0	0.9	1.0
25–34	1.1	1.0	0.5	1.5	**1.6**	1.36	0.9	1.4	0.7	1.3
35–44	2.0	2.4	1.8	2.5	2.1	2.39	2.2	2.8	2.4	**3.0**
45–54	4.8	3.4	4.7	4.7	4.1	3.31	4.1	3.3	4.5	**5.2**
55–64	8.5	5.2	8.4	7.5	7.2	7.58	5.4	**10.3**	7.4	7.5
65–74	9.1	7.5	8.6	9.1	9.6	9.02	8.2	7.2	**10.4**	7.8
75 +	14.0	13.5	14.8	11.4	**16.5**	13.5	11.9	13.8	11.3	12.9
***Usual Activities***										
16–24	**7.2**	4.8	5.4	5.7	4.1	3.1	5.1	3.5	5.5	6.3
25–34	**9.3**	7.2	5.3	5.6	6.3	5.0	8.3	5.9	5.9	8.8
35–44	10.5	9.4	6.3	9.0	9.1	8.1	9.7	**13.0**	8.7	10.4
45–54	15.5	12.7	14.4	13.7	12.3	11.4	11.7	**15.5**	13.0	13.3
55–64	**27.3**	20.5	19.9	22.2	20.2	20.9	20.2	24.4	17.6	16.1
65–74	**28.3**	20.9	24.3	21.9	24.7	24.5	21.4	25.7	20.9	17.5
75 +	38.6	36.9	35.2	29.9	36.7	36.3	33.1	**41.9**	27.9	30.4
***Pain/Discomfort***										
16–24	**22.1**	11.5	16.9	14.3	9.3	12.8	14.4	11.6	13.4	11.4
25–34	**21.4**	17.4	17.5	11.3	13.7	16.6	17.5	20.4	13.7	16.6
35–44	26.4	24.0	21.0	22.3	21.8	26.4	27.6	**33.0**	23.0	26.2
45–54	**36.1**	30.1	33.8	32.7	30.8	32.2	30.9	34.4	28.6	28.4
55–64	**45.3**	38.7	42.0	42.2	37.6	39.0	44.9	44.8	40.6	36.3
65–74	48.3	38.3	46.6	44.4	46.4	47.6	46.9	**52.6**	40.9	41.2
75 +	55.4	51.9	55.7	49.8	54.6	56.3	53.8	**61.3**	52.0	51.8
***Anxiety/Depression***										
16–24	15.3	11.4	9.6	13.7	9.5	11.8	**17.1**	15.4	12.8	15.5
25–34	15.2	16.5	13.9	13.0	13.3	13.9	18.2	**20.4**	12.9	17.9
35–44	20.2	15.5	15.4	15.9	15.2	15.6	20.0	**26.1**	17.1	15.8
45–54	23.2	15.1	18.7	15.8	19.5	17.4	22.3	**24.1**	19.7	18.0
55–64	21.8	17.5	16.7	19.6	17.9	15.6	22.5	**28.5**	18.8	18.5
65–74	**22.4**	14.5	14.8	12.7	13.8	15.6	15.0	20.3	14.7	12.4
75 +	23.4	20.4	18.3	12.2	16.9	18.1	20.9	**25.9**	17.6	15.9

Bold values indicate the highest value

**Table 3:** Age-stratified ANY problem rates (Women)

Women	1996	2003	2004	2005	2006	2008	2010	2011	2012	2014
***Mobility***										
16–24	**6.3**	4.2	2.7	4.3	3.9	4.0	1.9	4.8	4.9	4.7
25–34	6.5	4.2	7.4	5.6	4.9	6.0	5.8	6.0	4.0	**7.6**
35–44	8.7	8.4	8.3	9.0	7.4	8.2	9.2	**11.1**	9.1	9.9
45–54	14.3	15.7	15.8	15.2	15.5	16.9	18.1	**15.6**	15.1	13.8
55–64	26.5	23.8	26.2	23.3	23.9	22.9	23.6	**29.0**	22.1	21.0
65–74	34.4	33.1	32.5	31.3	34.6	37.4	32.4	**34.7**	29.0	26.6
75 +	53.2	57.6	52.3	54.1	55.3	55.2	56.6	**53.3**	51.3	49.6
***Self care***										
16–24	1.2	1.2	0.3	0.7	0.8	0.9	0.2	0.2	**1.6**	1.4
25–34	1.3	1.2	1.6	1.9	1.2	1.6	1.6	1.4	0.8	**2.0**
35–44	2.4	2.8	2.3	**3.3**	2.1	2.0	3.2	3.0	3.1	3.0
45–54	4.3	4.6	4.7	3.9	4.9	5.8	**6.8**	4.4	5.0	4.0
55–64	7.6	5.6	7.3	7.0	6.8	7.4	7.4	6.8	7.7	**8.7**
65–74	9.9	8.5	7.8	7.5	8.3	**11.6**	7.0	8.0	7.0	8.0
75 +	**17.8**	17.0	16.5	15.4	17.8	15.6	17.6	14.3	15.9	13.8
***Usual Activities***										
16–24	**10.3**	4.5	5.8	5.2	4.4	5.1	5.1	7.0	7.5	8.0
25–34	10.4	6.8	8.8	7.9	7.7	6.9	**10.7**	10.0	7.0	10.4
35–44	13.2	10.2	10.1	10.4	9.8	10.3	11.0	**15.3**	11.0	10.2
45–54	17.5	16.5	16.2	15.2	15.7	15.9	17.8	**19.3**	16.7	15.1
55–64	26.0	20.0	22.1	21.9	21.3	20.7	20.9	**27.6**	21.7	23.5
65–74	**30.6**	26.2	24.9	23.5	26.6	29.6	25.7	29.3	23.1	20.8
75 +	44.9	44.5	41.9	41.9	43.9	45.2	**46.2**	45.7	40.1	36.6
***Pain/Discomfort***										
16–24	**23.4**	14.0	14.0	12.7	14.7	16.4	13.3	21.3	15.4	18.4
25–34	**24.0**	16.9	19.3	17.1	16.4	19.1	19.8	20.7	16.8	17.8
35–44	30.9	23.3	24.9	25.2	23.2	23.6	25.9	**33.2**	25.5	24.9
45–54	39.4	34.6	36.7	36.3	36.8	35.5	36.4	**41.9**	34.3	34.4
55–64	50.8	43.4	48.0	47.1	43.4	44.8	43.6	**52.6**	48.0	43.8
65–74	54.7	53.1	55.4	52.3	54.5	55.7	49.3	**57.5**	52.8	46.9
75 +	58.1	61.9	63.1	63.9	60.2	65.0	64.3	**68.8**	63.4	63.6
***Anxiety/Depression***										
16–24	23.9	16.4	17.7	16.6	13.9	17.4	17.4	**25.6**	21.0	24.2
25–34	21.9	18.1	16.6	17.9	18.4	19.9	21.1	**24.7**	16.3	20.0
35–44	24.3	21.6	21.8	22.3	20.1	19.4	25.4	**28.7**	24.2	20.3
45–54	25.8	21.9	24.4	24.2	23.5	23.5	26.4	**30.5**	24.8	21.3
55–64	27.3	22.9	24.4	25.7	22.7	23.0	27.0	**31.8**	25.1	24.7
65–74	28.4	24.1	24.5	17.6	18.3	23.4	25.7	**29.5**	21.1	19.7
75 +	28.2	29.2	20.7	19.4	23.6	25.9	29.6	**31.9**	27.6	22.1

Bold values indicate the highest value

**Fig. 1:**
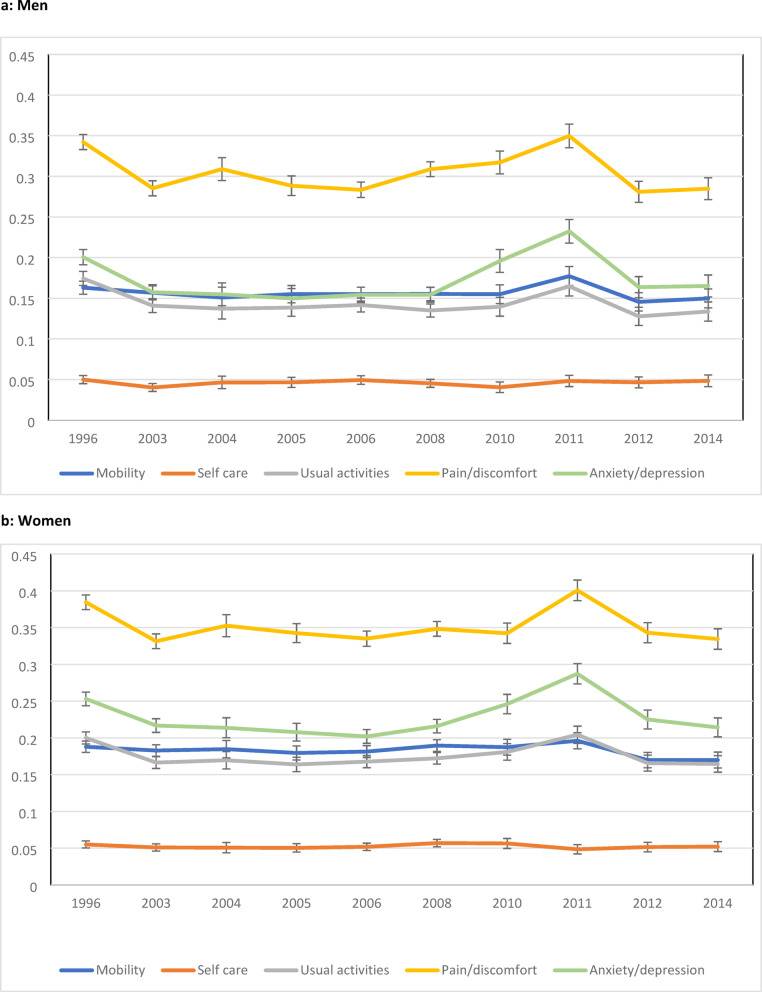
Standardised rates of reporting any problem in EQ-5D dimension

Age-standardised rates using HSE 1996 as the index year are shown in Fig. 1a and b. In men, the rates for Mobility and Usual Activities decline from 1996 and remain fairly constant, rising to a peak in 2011 before falling back. Anxiety/Depression levels initially follow a similar pattern but rise steeply between 2008 and 2011 with rates increasing from 15 to 23%. Pain/Discomfort rates fluctuate slightly but rise from 28% in 2006 to peak at 35% in 2011, falling back thereafter. A generally similar pattern is also evident for women.

The variations in EQ VAS over the 4 years are consistent with the findings indicated by the descriptive system with the mean EQ VAS in 2011 being significantly lower than that for all other years as can be seen in Table 4. The age and gender stratified EQ VAS scores exhibit a similar pattern. Variation in EQ VAS is statistically different for female respondents in all age groups, except the groups aged 75 + and for male respondents with the exception of men aged 65–74.

**Table 4:** Mean EQ-VAS scores stratified by age and gender

	2010		2011		2012		2014	
	*n* = 6,974		*n* = 7,246		*n* = 6,382		*n* = 6,490	
Mean EQ VAS	78.4 (SD 16.7)		74.4 (SD 17.6)		77.8 (SD 18.3)		78.0 (SD 18.4)	
	Mean	SD	Mean	SD	Mean	SD	Mean	SD
**Men**								
16–24	81.2	14.0	77.1	14.9	83.3	13.5	81.9	15.5
25–34	81.2	13.3	77.2	14.9	82.1	14.2	80.8	16.2
35–44	79.4	14.9	75.9	16.0	80.1	15.6	79.5	16.8
45–54	78.6	16.5	75.2	16.4	79.2	17.4	78.9	17.3
55–64	76.9	17.1	73.4	18.6	75.8	20.0	77.4	18.7
***65–74***	77.0	18.3	74.9	17.4	75.2	20.0	77.7	17.3
75 +	74.0	17.6	68.3	21.0	72.4	18.3	68.9	21.1
**Women**								
16–24	78.6	16.3	75.5	16.3	80.4	16.8	80.7	17.6
25–34	81.0	16.0	76.3	15.9	81.4	15.0	78.5	19.0
35–44	80.3	15.7	76.1	17.7	80.2	17.5	81.0	16.6
45–54	79.0	17.5	73.9	18.7	77.2	19.5	79.0	18.4
55–64	79.2	16.5	74.2	18.5	76.2	20.1	75.7	21.0
65–74	77.6	17.8	72.8	17.7	76.5	18.4	76.4	17.9
***75 +***	69.6	19.0	67.3	19.6	67.8	20.0	68.8	20.4

Bolditalic indicates non-significant differences. SD: standard deviation

EQ VAS can be interpreted as a measure of the respondent’s own value placed on their health status. Table 5 shows the mean EQ VAS ratings for the most frequently occurring EQ-5D-3L health states. These data are also shown graphically in Supplementary Material Fig. 2. Mean EQ VAS ratings for frequently occurring EQ-5D-3L health states. Taken together these health states account for some 90% of the HSE survey respondents. The most commonly encountered state is 11111 corresponding to full health indicating the absence of problems on all the EQ-5D-3L dimensions. In male respondents this proportion was 61.2% for HSE 2014, some 9 points higher than the proportion recorded for 2011; corresponding figures for women are lower, both being around 55.4% in 2014 and 46.2% in 2011. For both male and female respondents the mean value for the no problem health state (11111) is significantly different across the 4 years (ANOVA test p-value < 0.0001). Mean values for the remaining states reported by men are not significantly different, whereas for women several states are associated with mean EQ VAS values that are significantly different.

**Table 5:** Mean (Standard Deviation) EQ-VAS for most frequently occurring EQ-5D-3L health States

	2010			2011			2012			2014		
	N	Mean	SD	N	Mean	SD	N	Mean	SD	N	Mean	SD
**Men**												
***11111***	1,705	84.8	10.6	1,659	81.4	12.2	1,723	85.4	11.0	1,770	84.7	12.03
11121	365	78.6	12.8	352	77.0	13.4	342	78.5	11.5	301	79.2	13.8
11112	234	76.6	14.2	264	74.4	13.7	158	75.1	15.0	165	77.2	12.3
21121	112	75.5	13.6	94	75.9	13.4	90	72.8	16.2	91	72.8	13.9
21221	105	69.1	15.5	117	65.0	15.6	80	64.9	20.1	73	65.1	18.6
11122	95	75.4	11.7	130	72.5	13.6	65	70.6	15.2	83	71.5	14.8
21222	55	59.0	16.8	87	56.5	17.2	42	55.7	15.9	37	53.6	17.8
11221	35	75.4	13.5	58	72.4	14.5	34	75.2	10.6	29	72.0	17.7
11222	35	62.5	15.7	22	62.0	14.8	18	59.2	19.5	23	63.8	18.8
22222	28	50.7	13.5	32	51.4	20.3	26	46.0	16.4	23	46.0	14.2
21122	27	63.3	18.2	37	62.5	16.3	23	62.4	13.3	12	68.7	11.2
**Women**												
***11111***	2,057	85.5	12.0	1,881	82.1	12.6	1,873	85.7	11.5	1,995	85.3	12.7
***11121***	403	81.7	11.5	439	76.2	14.8	413	79.6	13.1	402	78.8	14.8
11112	361	77.8	15.0	385	75.2	15.6	278	77.4	14.8	272	77.4	15.9
11122	152	72.6	14.0	244	71.1	15.6	145	72.1	16.0	135	73.7	17.4
21221	147	66.6	14.5	184	66.1	17.3	119	62.8	15.8	132	64.4	15.9
21121	101	75.8	14.5	115	70.4	15.4	101	73.1	15.0	85	72.3	19.3
***21222***	97	61.7	15.9	125	59.4	14.3	81	53.7	17.7	73	59.8	18.4
11221	55	73.5	13.3	92	71.6	14.4	58	69.4	19.2	52	74.1	14.9
21122	51	70.0	15.5	47	63.5	18.3	30	64.8	15.5	32	68.0	16.4
***22222***	50	56.3	16.2	37	50.3	14.7	25	49.8	15.9	31	45.5	14.9
11222	43	63.7	15.1	66	62.6	13.7	38	56.1	16.0	33	61.1	23.2

Bolditalic cell indicates significant ANOVA test, *p* < 0.05

The results from logit regressions for each EQ-5D-3L dimension are shown in Table 6. The analysis excluded HSE 1996 data since some of the covariates were not collected that year. After adjusting for all covariates the year 2011 remains significantly different in each EQ-5D-3L dimension. This suggests that there are other factors influencing the higher rates of reporting problems in year 2011. The regression results also demonstrate that most of the covariates had statistically significant coefficients. For instance, individuals being older and female are more likely to report problems; having lower education and economic inactivity is associated with the likelihood of reporting more problems; those with higher total household income are likely to report fewer problems; those having more days with acute illness or having a long-standing illness/condition are more likely to report a problem. The deprivation index is also associated with reporting problems – the higher the index value, the more likely it is to report problems. The only exceptions are covariates of government office region and marital status, which did not show statistically significant associations in most EQ-5D-3L dimensions. Similar results are observed for EQ VAS, except in the case of older age, which did not show statistically significant influence.

**Table 6:** Results of logistic regression for each EQ-5D dimension (coefficients are shown as odd ratios) & results of OLS regression for EQ-VAS

	Mobility	Self care	Usual Activities	Pain/ Discomfort	Anxiety/ Depression	EQ-VAS
Female	0.95	0.85***	1.00	1.14***	1.27***	0.99***
	(0.027)	(0.037)	(0.029)	(0.023)	(0.027)	(0.196)
Age	1.02***	1.03***	1.00	1.05***	1.04***	-0.06
	(0.005)	(0.008)	(0.005)	(0.004)	(0.004)	(0.041)
Age squared	1.00***	1.00	1.00***	1.00***	1.00***	0.000
	(0.000)	(0.000)	(0.000)	(0.000)	(0.000)	(0.000)
Married/civil partner	1.00	1.01	1.11**	1.23***	0.80***	0.82**
	(0.050)	(0.078)	(0.057)	(0.044)	(0.029)	(0.359)
Separated	1.12	0.97	1.17	1.04	1.38***	0.35
	(0.103)	(0.139)	(0.114)	(0.072)	(0.093)	(0.718)
Divorced	1.03	1.06	1.08	1.17***	1.10**	-0.93
	(0.065)	(0.098)	(0.070)	(0.056)	(0.052)	(0.540)
Widowed	1.06	0.95	1.09	1.11**	1.02	-0.96
	(0.067)	(0.086)	(0.073)	(0.057)	(0.054)	(0.622)
Cohabitee	0.99	0.99	1.09	1.21***	0.96	-0.10
	(0.064)	(0.107)	(0.069)	(0.052)	(0.041)	(0.410)
In employment	1.24**	0.98	1.17	1.09	1.45***	-1.41**
	(0.113)	(0.202)	(0.108)	(0.063)	(0.075)	(0.519)
Unemployed	1.21***	2.81***	1.37***	0.95	0.99	0.12
	(0.055)	(0.230)	(0.068)	(0.034)	(0.039)	(0.408)
Retired	2.14***	5.84***	2.64***	1.13***	1.69***	-3.96***
	(0.087)	(0.426)	(0.108)	(0.036)	(0.050)	0.386
Higher education	1.32***	0.93	1.12**	1.27***	0.91**	-0.09
	(0.071)	(0.093)	(0.063)	(0.047)	(0.037)	(0.333)
A level equivalent	1.23***	1.11	1.09	1.21***	0.98	-0.37
	(0.070)	(0.115)	(0.062)	(0.044)	(0.037)	(0.306)
O level equivalent	1.26***	1.12	1.11**	1.34***	1.01	-0.64**
	(0.062)	(0.097)	(0.055)	(0.043)	(0.035)	(0.286)
Other grade equivalent	1.49***	1.26**	1.30***	1.47***	1.06	-2.73***
	(0.107)	(0.143)	(0.095)	(0.076)	(0.056)	(0.576)
Other qualifications	1.40***	1.18	1.11	1.55***	1.08	-0.36
	(0.120)	(0.159)	(0.101)	(0.106)	(0.081)	(0.883)
No qualification	1.67***	1.40***	1.36***	1.61***	1.10**	-2.71***
	(0.080)	(0.116)	(0.068)	(0.056)	(0.040)	(0.386)
Income 10,400 to under 20,800	0.98	1.23***	1.01	0.91***	0.82***	1.80***
	(0.037)	(0.064)	(0.041)	(0.030)	(0.026)	(0.440)
Income 20,800 to under 33,800	0.82***	0.97	0.87***	0.82***	0.69***	3.59***
	(0.039)	(0.070)	(0.043)	(0.031)	(0.026)	(0.465)
Income 33,800 to under 52,000	0.81***	0.79**	0.80***	0.74***	0.64***	4.01***
	(0.045)	(0.080)	(0.046)	(0.031)	(0.027)	(0.486)
Income over 52,000	0.75***	0.74***	0.74***	0.69***	0.57***	4.22***
	(0.044)	(0.082)	(0.045)	(0.030)	(0.026)	(0.490)
Has non-limiting illness	0.13***	0.10***	0.10***	0.25***	0.47***	12.66***
	(0.005)	(0.009)	(0.004)	(0.007)	(0.014)	(0.369)
No limiting illness	0.07***	0.06***	0.06***	0.12***	0.30***	16.01***
	(0.002)	(0.005)	(0.002)	(0.003)	(0.008)	(0.340)
1–3 days of acute illness	1.59***	1.34***	2.36***	1.89***	1.38***	-2.21***
	(0.090)	(0.117)	(0.129)	(0.083)	(0.060)	(0.491)
4–6 days of acute illness	2.09***	1.80***	3.14***	2.33***	1.68***	-5.24***
	(0.140)	(0.162)	(0.218)	(0.140)	(0.093)	(0.772)
7–13 days of acute illness	2.67***	2.59***	3.68***	2.51***	1.79***	-7.94***
	(0.171)	(0.194)	(0.242)	(0.144)	(0.091)	(0.765)
Full 2 weeks of acute illness	4.00***	3.20***	7.13***	3.85***	1.99***	-11.74***
	(0.188)	(0.166)	(0.357)	(0.182)	(0.073)	(0.574)
IMD score 8.32->13.74	1.11**	1.11	1.10**	1.07**	0.99	-0.37
	(0.048)	(0.082)	(0.050)	(0.033)	(0.034)	(0.295)
IMD score 13.74->21.22	1.23***	1.26***	1.11**	1.13***	1.03	-1.05**
	(0.054)	(0.093)	(0.051)	(0.036)	(0.036)	(0.317)
IMD score 21.22->34.42	1.36***	1.48***	1.27***	1.16***	1.09**	-1.22***
	(0.062)	(0.109)	(0.060)	(0.039)	(0.039)	(0.341)
IMD score 34.42->85.46 [most deprived]	1.55***	1.69***	1.31***	1.29***	1.10**	-2.28***
	(0.076)	(0.129)	(0.067)	(0.047)	(0.042)	(0.389)
North West	0.98	1.05	1.03	1.04	1.01	-0.42
	(0.059)	(0.088)	(0.064)	(0.048)	(0.048)	(0.478)
Yorkshire and Humber	0.83***	0.90	0.89	0.90**	0.96	-0.14
	(0.053)	(0.081)	(0.059)	(0.045)	(0.048)	(0.506)
East Midlands	0.92	1.00	0.89	0.98	0.98	0.76
	(0.060)	(0.095)	(0.061)	(0.049)	(0.051)	(0.496)
West Midlands	1.00	1.08	0.97	1.06	0.98	0.10
	(0.063)	(0.097)	(0.064)	(0.051)	(0.049)	(0.500)
East of England	0.97	1.04	0.93	1.01	1.03	0.52
	(0.061)	(0.096)	(0.060)	(0.049)	(0.051)	(0.471)
London	0.94	1.07	0.93	1.10	1.04	-0.04
	(0.063)	(0.103)	(0.067)	(0.056)	(0.053)	(0.504)
South East	0.95	0.85	0.86**	1.00	0.99	0.12
	(0.058)	(0.078)	(0.055)	(0.046)	(0.047)	(0.444)
South West	0.93	0.88	0.87**	1.00	0.98	0.80
	(0.060)	(0.085)	(0.059)	(0.050)	(0.050)	(0.581)
2004.year	0.96	1.08	1.01	1.16***	0.98	--
	(0.057)	(0.097)	(0.061)	(0.052)	(0.047)	--
2005.year	0.94	0.98	0.96	1.10**	0.95	--
	(0.048)	(0.077)	(0.053)	(0.044)	(0.041)	--
2006.year	1.01	1.14	1.05	1.09**	0.98	--
	(0.048)	(0.086)	(0.053)	(0.039)	(0.038)	--
2008.year	1.14***	1.23***	1.10**	1.25***	1.05	--
	(0.054)	(0.089)	(0.055)	(0.044)	(0.040)	--
2010.year	1.14**	1.18	1.19***	1.33***	1.37***	--
	(0.065)	(0.103)	(0.072)	(0.056)	(0.061)	--
2011.year	1.48***	1.22**	1.80***	1.86***	1.81***	-4.28***
	(0.084)	(0.111)	(0.106)	(0.078)	(0.076)	(0.278)
2012.year	1.10	1.35***	1.17***	1.36***	1.25***	-1.20***
	(0.064)	(0.119)	(0.070)	(0.057)	(0.056)	(0.289)
2014 year	1.09	1.39***	1.19***	1.27***	1.18***	-0.98**
	(0.064)	(0.124)	(0.074)	(0.056)	(0.054)	(0.295)
_cons	0.06***	0.01***	0.15***	0.17***	0.20***	68.13***
	(0.010)	(0.002)	(0.023)	(0.019)	(0.023)	(1.158)
N	68,210	68,210	68,210	68,210	68,210	16,437

p-values : * *p* < 0.1 ** *p* < 0.05, *** *p* < 0.01; standard error in parenthesis

All these analyses were repeated with survey weights provided in the HSE data from 2003 onwards (1996 data was excluded as no weights were available). Similar results as those based on the above unweighted analyses were produced. The results from the weighted analyses are available upon request from the author. Additionally, norms based on the year 2014 by age and gender, weighted by individual weights, are provided in Supplementary Material Tables 2–3.

The primary objective of this study was the analysis of self-reported health status as directly observed using EQ-5D-3L and has been limited to the descriptive profile (self-classifier) and EQ VAS ratings. However, it is recognised that these same data can be represented as a summary score (EQ-5D index score), based on separately obtained social preference weights [13]the essential difference being that these weights represent the values of the general public rather than those of the respondent themselves.

The pattern of variation based on respondent self-report as already presented was largely replicated in the EQ-5D index scores. Supplementary Material Table 4 presents observed mean index scores for men and women. Supplementary Material Figure 3 Standardized mean EQ-5D index scores presents age-standardised mean index score using HSE 1996 as the index year.

## Discussion

Monitoring self-reported health status in the general population over time requires repeated observation using a standardised measure. Since there is inevitably a lag between executing a national population survey and the publication of derived data on health status, end-users are forced to assume that older reported data adequately represents contemporary values. For some 2 decades, the Health Survey for England included EQ-5D-3L, thereby creating probably the longest cross-sectional record of such information. This accumulation of self-reported data provides an opportunity to examine the extent of any variation in health status as defined by EQ-5D over time.

EQ-5D is essentially a self-classification system based on 5 dimensions that can be separately reported or combined to identify a single health state or profile. Results reported here suggest 4 patterns across the study period with the first showing a fall in problem rates between 1996 and 2003 followed by a degree of stability until 2006. Thereafter, rates increase to a peak in 2011 before falling back in 2014 to rates that approximate those for 2003. Viewed separately, rates for some dimensions display a degree of similarity, with apparent covariation involving Anxiety/Depression, Pain/Discomfort in particular - by contrast Self-care appears to flatline throughout. However, were data for the intervening years not available, it seems reasonable to suggest that self-reported health status in 2014 would be regarded as being unchanged from levels seen a decade earlier.

Of particular interest is the pattern that can be seen from 2006. Problem rates for Anxiety/Depression amongst women rise significantly in each of the following surveys, to a peak in 2011 with a rate that is 40% higher. A similar pattern can be seen for Pain/Discomfort with an increase of some 20%. Corresponding increases in these dimension problem rates in men are 50% and 34% respectively. Problem rates post-2011 fall for all dimensions with the exception of Self-care which more or less flatlines across the entire study period.

Interpreting these observations presents an interesting challenge. Seemingly large increases in self-reported problems, consistent with a reduced level of health status can be seen in 2 key EQ-5D dimensions. Accounting for this remarkable difference is not an easy task, and in the first instance one would need to establish whether material alterations have been made to the survey methodology, its sampling and interview protocols. On the face of it, this seems an unlikely proposition as no methodological changes appear to have been published. However, examination of the self-completed booklets used in the 2011 survey reveal that a set of questions asking about attitudes to personal health and lifestyle were administered before EQ-5D-3L, whereas in previous years EQ-5D-3L preceded it. Even if this isolated alteration had somehow influenced the 2011 responses, it hardly accounts for the trend seen prior to that date. HSE surveys are widely accepted as being representative of the general population. In the absence of contra indications, it seems reasonable to infer that fluctuations in self-reported problems seen in the present study are unlikely to be associated with methodological changes or operational survey flaws.

If these observed patterns are non-artefactual, then other factors need to be considered such as economic, social or other system-wide shocks. The economic recession in 2008 is an obvious candidate, having prolonged and widespread effects. A 2014 WHO report [14] published following that financial crisis acknowledged that *economic shocks pose a threat to health and health system performance;* it went on to note that *mental health has been most sensitive to economic changes so far*, and that the *full scale of the effects of the crisis on health may not be apparent for years*. [p37]. If the otherwise anomalous results reported here in the period leading up to 2011 prove to be consistent with society-wide shocks, then this suggests an enhanced role for EQ-5D in monitoring population health. The peak seen in 2011 might, in part, be accentuated by the widespread civil unrest witnessed in that year, with several deaths and more than 3,000 arrests.

The findings based on EQ VAS are far less conclusive. Limited data availability across a shorter time horizon makes it difficult to draw firm conclusions regarding temporal variation. However, a somewhat similar pattern was observed in EQ VAS for 2011, which was lower than other years, indicating poorer health status. Again, this could be either an instrumental effect in completing the EQ-5D-3L questionnaire or the influence of other social/environmental factors. Despite the truncated data it was possible to chart the mean EQ VAS score over time for a limited number of health states. This allows for the comparison of respondents with the same profile of self-classifier responses. Amongst participants in full health state, that is reporting no problems on any of the 5 dimensions, the mean EQ VAS scores for men in the years 2010, 2012 and 2014 was 85.0, a figure some 4% higher than in 2011. This pattern holds similarly for women in full health.

Interestingly, in the HSE data the self–assessed 5-point rating scale remained relatively constant over time. (Data is shown in Supplementary Material Fig. 4. Standardized rates of self-assessed 5-point rating scale (self-reported health item)) The long-term rates of those reporting their health as being Bad or Very Bad, remain virtually unchanged. Such fluctuations as do occur can be seen for respondents reporting Excellent/Very Good health status. A discussion on the self–assessed 5-point rating scale is beyond the scope of the current manuscript. However, interested audience can refer to other studies [17] for further exploration on this measure.

There is an apparent dearth of published studies that explore temporal variation in self-rated EQ-5D. Longitudinal data collected over a 5-year period from 1999 amongst patients in two general practices in south Manchester [15] showed that after adjusting for potential confounders both the EQ-5D index score and EQ VAS score declined significantly. Deterioration in health-related quality of life was similarly observed in a Swedish study, where EQ-5D-3L data were collected in cross-sectional surveys from a representative sample of the Stockholm County population over a 4-year period [16] from 1998. Indications in the present study run counter to this and are more suggestive of longer-term stability. Were one to apply a similar 5-year time frame to the HSE data reported here, it would be possible to conclude that self-rated health status remained stable over the 5 years after 2003 and again after 2008. The changes demonstrated in the present study were only detected because standard EQ-5D data were available from repeated HSE surveys.

Ultimately, if the data reported in this study were restricted only to a comparison of population health in the years 2003 and 2014, then it would be difficult to avoid the conclusion that nothing had changed. Such a finding could disappoint those who might have anticipated an improvement, given the vast commitment of healthcare resources over that period, and health policy initiatives at local and national levels. Over this same period, average life expectancy has increased from 78.4 to 81.3 years, so that an alternative indicator [18]such as disability-free life expectancy (DFLE) that combines these two components, could be more informative.

One question that remains to be answered in measuring population health is what constitutes a “significant” variation? This applies to all measures of health-related quality of life, not only to EQ-5D. If we observe any differences over (short) periods of time, then how large do those differences have to be before we regard them as “significant”? This is not purely a statistical matter, rather it is context specific and depends largely upon the type of decision that is being informed.

Were significant variations to be identified in general population data, then this might have a material effect on long-running clinical trials or in the analysis of registry data, especially since contextual variables associated with such variation are likely to be unrecorded. In population studies, the choice of a comparator period may become a sensitive matter, especially if a selection can be made from across a number of years.

Some limitations to the current study must be acknowledged. Although the HSE surveys were all conducted by the same national agency with the same focus, over time the survey underwent changes in the questionnaire resulting in issues of (dis)continuity in the collection of individual and household variables. For instance, social economic class variables were collected in 1996 but not from 2010 onwards, whereas index of multiple deprivation was not collected in 1996 data. Local government boundaries also changed so that some geographical areas are no longer strictly comparable. Such changes impose difficulty in making a comparison over the study. Similarly, sampling weights are only available from 2003 onwards. Notably too, the HSE survey does not include institutionalized individuals, such as patients in hospitals or pensioners in retirement homes.

The current study reports on the latest available English population EQ-5D-3L data, however, these data are more than 10 years old and more recent HSE surveys are based on the revised 5L version of EQ-5D. So as to make the best use of such historic population data a mechanism for transforming the 3L descriptive system into its corresponding 5L equivalent will be needed. Since the EQ VAS remains common to both 3L and 5L forms of EQ-5D, it is likely that this will play a central role in determining that equivalence. Perhaps one lesson learned from the current study is the importance of collecting health-related quality of life data on a regular and continuous basis, using a single standard metric thereby creating the opportunity of observing potential temporal change as well as providing much needed up-to-date normative data.

## Conclusion

The study demonstrates the stability of EQ-5D responses over time in HSE data from 1996 to 2014. However, there is evidence of periodic deterioration in population health status notably in the years immediately after 2007. Understanding this apparent decline is important, firstly to identify association with possible explanatory variables, but also to signal the need for caution when using EQ-5D reference data from this period. The study demonstrates the importance of maintaining national surveys of the general population, with regular collection of health status data using a standardised measure of health-related quality of life. Without such a national resource, our capacity to monitor long-term trends in population health risks being fatally compromised.

## Supplementary Information

Below is the link to the electronic supplementary material.

Supplementary Material 1

## Data Availability

The main data source for the current study is Health Survey for England, available at: https://www.ucl.ac.uk/hssrg/studies/hse.
