# Supplementary material for: Self-reported health status in the general population over 2 decades: variation in EQ-5D-3L in Health Survey for England
Source: BMC Public Health. 2025 Sep 30;25:3207. doi: 10.1186/s12889-025-24238-2 (PMC12487048; doi:10.1186/s12889-025-24238-2)
Supplement: Supplementary file 1 — Supplementary Material 1 [file 12889_2025_24238_MOESM1_ESM.docx]

**Supplementary Material**

**Figure 1. Average height over time**

The figure presents a stable trend of average height of adult populations aged over 16 in the HSE data over time. The slight decrease in year 2005 is due to over-sample of aged population in that year.

**Figure 2: Mean EQ-VAS ratings for frequently occurring EQ-5D-3L health states**

**Figure 2a: Men**

**Figure 2b: Women**

The figures present the average EQ VAS scores for frequently occurring health states (defined as >50 observations in one of observed gender & year categories) from the HSE 2010, 2011, 2012 and 2014 data.

**Figure 3. Standardised mean EQ-5D index scores**


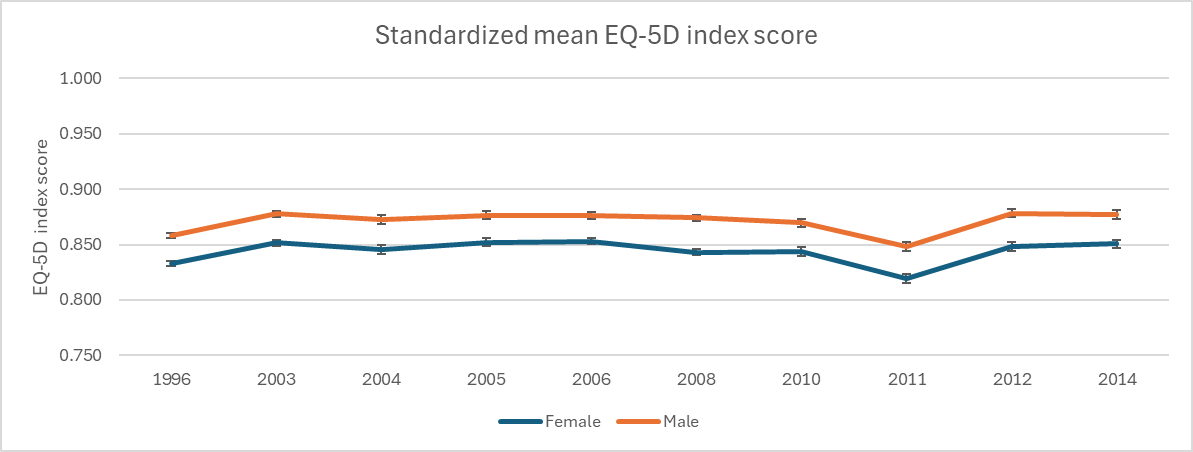


The figures present the standardized mean EQ-5D index score in the HSE data across indicated years. The standardization is based on the age distribution of the 1996 population.

**Figure 4. Standardised rates of self-assessed 5-point rating scale (self-reported health item)**

**Figure 4a. Men**


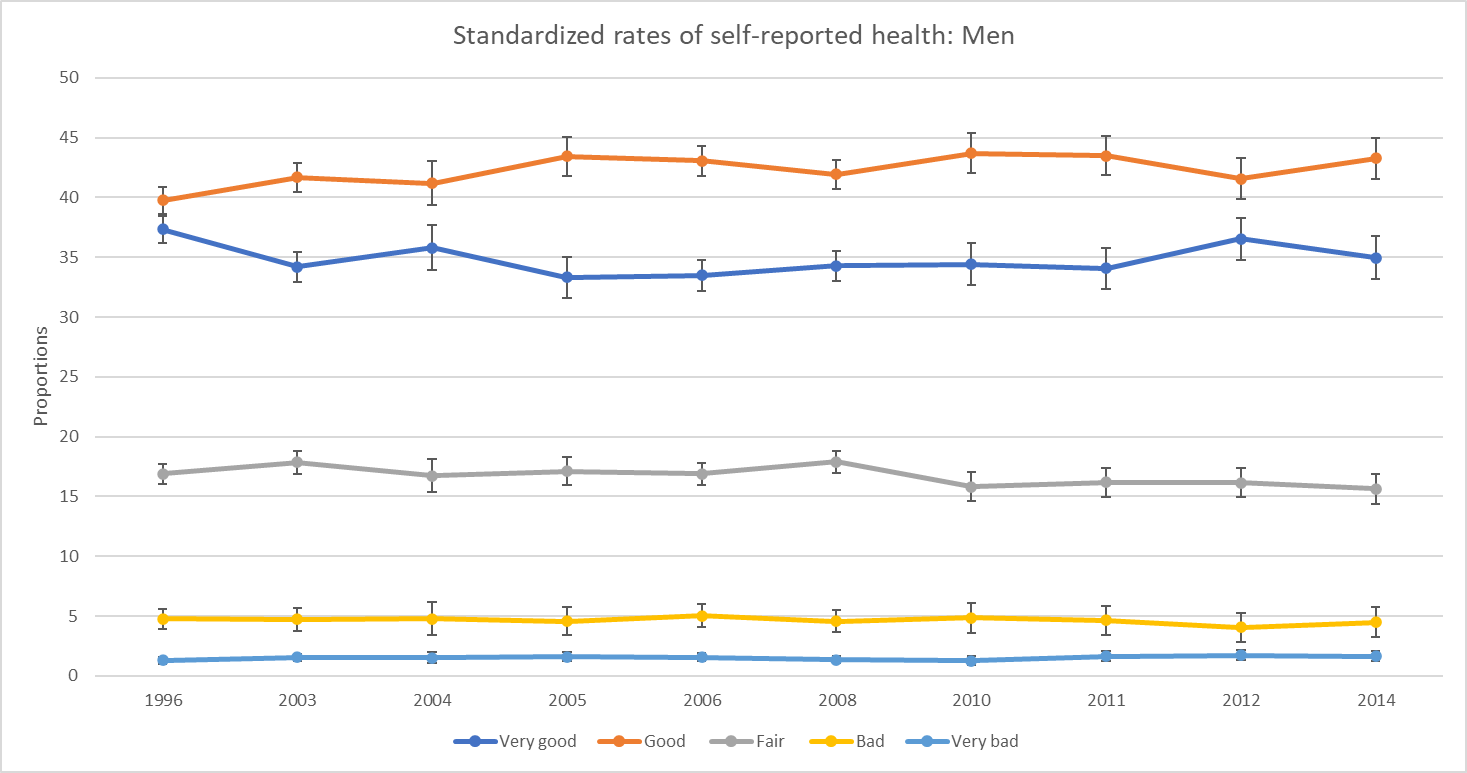


**Figure 4b. Women**


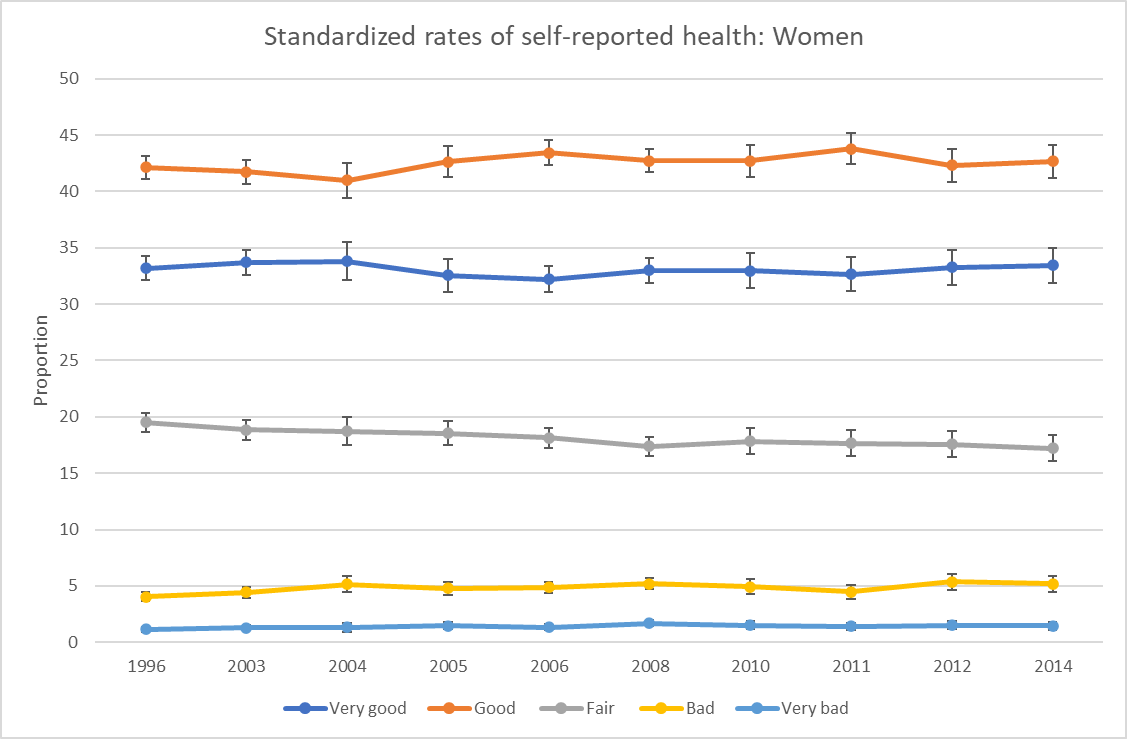


The Figures present the standardized rates of responses in the self-assessed 5-point rating scale (self-reported health [SRH] item) in the HSE data across indicated years. The standardisation is based on the age distribution of the 1996 population. The Figures suggest a stable trend for self-reported health as measured by the SRH item over time.

**Table 1: Observed distribution of problem rates for EQ-5D dimensions (% rates) in the HSE data**

|  | HSE | | | | | | | | | |
| --- | --- | --- | --- | --- | --- | --- | --- | --- | --- | --- |
|  | 1996 | 2003 | 2004 | 2005 | 2006 | 2008 | 2010 | 2011 | 2012 | 2014 |
| **Mobility** |  |  |  |  |  |  |  |  |  |  |
| no problems | 82.34 | 82.57 | 81.94 | 77.37 | 81.90 | 81.44 | 81.33 | 79.78 | 82.55 | 82.67 |
| some problems | 17.55 | 17.39 | 17.89 | 22.55 | 17.96 | 18.38 | 18.54 | 20.07 | 17.27 | 17.15 |
| confined to bed | 0.11 | 0.04 | 0.16 | 0.08 | 0.14 | 0.18 | 0.14 | 0.15 | 0.18 | 0.18 |
| *ANY problem* | *17.66* | *17.43* | *18.05* | *22.63* | *18.1* | *18.56* | *18.68* | *20.22* | *17.45* | *17.33* |
| **Self care** |  |  |  |  |  |  |  |  |  |  |
| no problems | 94.73 | 95.32 | 94.75 | 93.61 | 94.56 | 94.47 | 94.65 | 94.77 | 94.52 | 94.52 |
| some problems | 4.85 | 4.46 | 4.89 | 5.97 | 5.03 | 5.19 | 5.14 | 4.90 | 5.17 | 5.17 |
| unable to ^b^ | 0.42 | 0.23 | 0.36 | 0.42 | 0.41 | 0.35 | 0.20 | 0.33 | 0.32 | 0.31 |
| *ANY problem* | *5.27* | *4.69* | *5.25* | *6.39* | *5.44* | *5.54* | *5.34* | *5.23* | *5.49* | *5.48* |
| **Usual Activities** |  |  |  |  |  |  |  |  |  |  |
| no problems | 81.16 | 84.29 | 83.66 | 81.06 | 83.58 | 83.53 | 82.90 | 80.26 | 84.04 | 84.18 |
| some problems | 16.30 | 13.81 | 14.59 | 16.56 | 14.58 | 14.52 | 15.49 | 17.73 | 14.48 | 14.42 |
| unable to ^c^ | 2.54 | 1.90 | 1.75 | 2.39 | 1.85 | 1.95 | 1.61 | 2.01 | 1.48 | 1.40 |
| *ANY problem* | *18.84* | *15.71* | *16.34* | *18.95* | *16.43* | *16.47* | *17.1* | *19.74* | *15.96* | *15.82* |
| **Pain/Discomfort** |  |  |  |  |  |  |  |  |  |  |
| No problems | 63.47 | 68.60 | 65.33 | 62.40 | 67.55 | 65.74 | 65.38 | 60.65 | 66.47 | 67.20 |
| moderate | 33.06 | 27.76 | 30.44 | 33.24 | 28.66 | 30.09 | 30.93 | 34.46 | 29.63 | 28.98 |
| extreme | 3.47 | 3.64 | 4.24 | 4.35 | 3.79 | 4.17 | 3.68 | 4.90 | 3.91 | 3.82 |
| *ANY problem* | *36.53* | *31.4* | *34.68* | *37.59* | *32.45* | *34.26* | *34.61* | *39.36* | *33.54* | *32.80* |
| **Anxiety/Depression** |  |  |  |  |  |  |  |  |  |  |
| not | 77.08 | 80.93 | 80.95 | 82.24 | 81.77 | 81.08 | 77.41 | 73.41 | 79.94 | 80.76 |
| moderately | 21.06 | 17.39 | 17.29 | 16.20 | 16.34 | 17.16 | 20.55 | 24.08 | 17.84 | 17.06 |
| extremely | 1.85 | 1.68 | 1.77 | 1.56 | 1.89 | 1.76 | 2.03 | 2.51 | 2.22 | 2.17 |
| *ANY problem* | *22.91* | *19.07* | *19.06* | *17.76* | *18.23* | *18.92* | *22.58* | *26.59* | *20.06* | *19.24* |

^b^ to wash and dress; ^c^ to perform Usual Activities

**Table 2. HSE 2014 Norm: distribution of problem rates for EQ-5D dimensions (% rates)**

|  | **Overall** | | | | | | | **Male** | | | | | | | **Female** | | | | | | |
| --- | --- | --- | --- | --- | --- | --- | --- | --- | --- | --- | --- | --- | --- | --- | --- | --- | --- | --- | --- | --- | --- |
| Age group | 16-24 | 25-34 | 35-44 | 45-54 | 55-64 | 65-74 | 75 + | 16-24 | 25-34 | 35-44 | 45-54 | 55-64 | 65-74 | 75 + | 16-24 | 25-34 | 35-44 | 45-54 | 55-64 | 65-74 | 75 + |
| **Mobility** |  |  |  |  |  |  |  |  |  |  |  |  |  |  |  |  |  |  |  |  |  |
| no problems | 95.28 | 93.80 | 90.27 | 86.88 | 79.78 | 74.11 | 52.91 | 95.09 | 94.73 | 90.62 | 88.02 | 80.59 | 74.80 | 55.30 | 95.48 | 92.94 | 89.93 | 85.76 | 79.03 | 73.48 | 51.04 |
| some problems | 4.50 | 6.03 | 9.67 | 12.92 | 20.12 | 25.79 | 46.73 | 4.76 | 5.08 | 9.38 | 11.69 | 19.22 | 24.99 | 44.06 | 4.24 | 6.91 | 9.94 | 14.12 | 20.97 | 26.52 | 48.82 |
| confined to bed | 0.22 | 0.17 | 0.06 | 0.21 | 0.09 | 0.10 | 0.36 | 0.15 | 0.19 | 0.00 | 0.29 | 0.20 | 0.21 | 0.64 | 0.28 | 0.15 | 0.13 | 0.13 | 0.00 | 0.00 | 0.14 |
| **Self care** |  |  |  |  |  |  |  |  |  |  |  |  |  |  |  |  |  |  |  |  |  |
| no problems | 98.95 | 98.66 | 97.08 | 95.61 | 91.84 | 92.36 | 86.84 | 99.17 | 99.09 | 97.26 | 95.09 | 92.52 | 92.32 | 87.51 | 98.72 | 98.27 | 96.91 | 96.13 | 91.21 | 92.40 | 86.32 |
| some problems | 0.83 | 1.20 | 2.76 | 4.18 | 7.68 | 7.23 | 12.74 | 0.67 | 0.91 | 2.48 | 4.63 | 6.84 | 7.47 | 11.86 | 1.00 | 1.47 | 3.02 | 3.74 | 8.47 | 7.01 | 13.43 |
| unable to ^b^ | 0.22 | 0.14 | 0.16 | 0.21 | 0.47 | 0.41 | 0.42 | 0.15 | 0.00 | 0.25 | 0.29 | 0.64 | 0.21 | 0.63 | 0.28 | 0.26 | 0.07 | 0.13 | 0.32 | 0.60 | 0.25 |
| **Usual Activities** |  |  |  |  |  |  |  |  |  |  |  |  |  |  |  |  |  |  |  |  |  |
| no problems | 93.16 | 91.48 | 89.95 | 85.95 | 80.30 | 81.29 | 66.80 | 94.08 | 92.56 | 90.08 | 87.39 | 84.48 | 82.75 | 70.24 | 92.21 | 90.48 | 89.82 | 84.54 | 76.40 | 79.97 | 64.11 |
| some problems | 6.65 | 8.21 | 9.25 | 13.03 | 17.27 | 17.06 | 29.07 | 5.54 | 7.22 | 9.04 | 10.96 | 13.06 | 15.09 | 25.02 | 7.79 | 9.13 | 9.44 | 15.06 | 21.20 | 18.84 | 32.23 |
| unable to ^c^ | 0.19 | 0.31 | 0.81 | 1.01 | 2.43 | 1.65 | 4.13 | 0.38 | 0.22 | 0.88 | 1.65 | 2.46 | 2.16 | 4.74 | 0.00 | 0.39 | 0.74 | 0.39 | 2.41 | 1.19 | 3.66 |
| **Pain/Discomfort** |  |  |  |  |  |  |  |  |  |  |  |  |  |  |  |  |  |  |  |  |  |
| No problems | 86.26 | 84.07 | 74.74 | 68.33 | 60.12 | 55.99 | 41.87 | 89.50 | 84.70 | 74.51 | 71.74 | 64.17 | 58.82 | 49.12 | 82.92 | 83.49 | 74.97 | 64.99 | 56.36 | 53.42 | 36.22 |
| moderate | 13.07 | 14.73 | 22.77 | 28.79 | 33.41 | 38.34 | 51.13 | 10.50 | 14.19 | 23.28 | 25.31 | 31.18 | 36.86 | 45.75 | 15.71 | 15.22 | 22.28 | 32.19 | 35.49 | 39.68 | 55.33 |
| extreme | 0.67 | 1.20 | 2.49 | 2.89 | 6.47 | 5.68 | 7.00 | 0.00 | 1.11 | 2.21 | 2.95 | 4.66 | 4.32 | 5.13 | 1.37 | 1.29 | 2.75 | 2.82 | 8.15 | 6.91 | 8.45 |
| **Anxiety/Depression** |  |  |  |  |  |  |  |  |  |  |  |  |  |  |  |  |  |  |  |  |  |
| not | 80.39 | 81.17 | 81.91 | 81.04 | 78.93 | 84.00 | 80.43 | 84.93 | 81.96 | 84.05 | 82.84 | 82.44 | 88.02 | 84.27 | 75.70 | 80.44 | 79.85 | 79.28 | 75.66 | 80.35 | 77.43 |
| moderately | 17.33 | 17.14 | 15.84 | 16.91 | 18.49 | 14.41 | 17.95 | 13.30 | 17.05 | 13.74 | 15.12 | 15.33 | 10.76 | 15.10 | 21.48 | 17.22 | 17.85 | 18.65 | 21.43 | 17.71 | 20.17 |
| extremely | 2.28 | 1.69 | 2.25 | 2.05 | 2.58 | 1.60 | 1.63 | 1.77 | 0.99 | 2.20 | 2.04 | 2.23 | 1.22 | 0.63 | 2.81 | 2.35 | 2.30 | 2.07 | 2.91 | 1.94 | 2.40 |

b: to wash and dress; c: to perform Usual Activities

**Table 3. HSE 2014 Norm: index and EQ VAS scores**

|  | Age |  |  |  |  |  |  |
| --- | --- | --- | --- | --- | --- | --- | --- |
| **Index score *** | 16-24 | 25-34 | 35-44 | 45-54 | 55-64 | 65-74 | 75 above |
| **Overall** |  |  |  |  |  |  |  |
| Mean | 0.925 | 0.920 | 0.890 | 0.871 | 0.820 | 0.823 | 0.747 |
| SD | 0.155 | 0.166 | 0.205 | 0.215 | 0.273 | 0.249 | 0.262 |
| **Male** |  |  |  |  |  |  |  |
| Mean | 0.942 | 0.927 | 0.894 | 0.878 | 0.843 | 0.839 | 0.776 |
| SD | 0.131 | 0.147 | 0.202 | 0.221 | 0.253 | 0.232 | 0.251 |
| **Female** |  |  |  |  |  |  |  |
| Mean | 0.907 | 0.913 | 0.886 | 0.864 | 0.798 | 0.808 | 0.723 |
| SD | 0.174 | 0.181 | 0.207 | 0.209 | 0.289 | 0.263 | 0.268 |
|  | Age |  |  |  |  |  |  |
| **EQ VAS** | 16-24 | 25-34 | 35-44 | 45-54 | 55-64 | 65-74 | 75 above |
| **Overall** |  |  |  |  |  |  |  |
| Mean | 81.39 | 80.05 | 80.37 | 78.94 | 76.61 | 77.21 | 69.12 |
| SD | 16.42 | 16.96 | 16.40 | 17.88 | 19.88 | 17.52 | 20.51 |
| **Male** |  |  |  |  |  |  |  |
| Mean | 82.15 | 81.02 | 79.74 | 78.90 | 77.73 | 77.88 | 69.36 |
| SD | 14.78 | 15.56 | 16.50 | 17.37 | 18.61 | 17.27 | 20.76 |
| **Female** |  |  |  |  |  |  |  |
| Mean | 80.61 | 79.13 | 80.99 | 78.98 | 75.53 | 76.56 | 68.93 |
| SD | 17.93 | 18.18 | 16.30 | 18.39 | 21.01 | 17.76 | 20.36 |

* Based on the UK 3L value set. Reference: Dolan P. Modeling valuations for EuroQol health states. Med Care 1997 Nov;35(11):1095-108.

**Table 4. Observed mean EQ-5D index scores* in HSE data**

| **Male** | 1996 | 2003 | 2004 | 2005 | 2006 | 2008 | 2010 | 2011 | 2012 | 2014 |
| --- | --- | --- | --- | --- | --- | --- | --- | --- | --- | --- |
| *16-24* | 0.913 | 0.949 | 0.937 | 0.940 | 0.956 | 0.947 | 0.930 | 0.945 | 0.940 | 0.938 |
| *25-34* | 0.916 | 0.918 | 0.934 | 0.938 | 0.934 | 0.930 | 0.922 | 0.914 | 0.939 | 0.921 |
| *35-44* | 0.895 | 0.905 | 0.916 | 0.902 | 0.909 | 0.897 | 0.893 | 0.858 | 0.907 | 0.892 |
| *45-54* | 0.852 | 0.881 | 0.854 | 0.868 | 0.868 | 0.877 | 0.865 | 0.852 | 0.865 | 0.875 |
| *55-64* | 0.800 | 0.830 | 0.820 | 0.816 | 0.831 | 0.831 | 0.813 | 0.793 | 0.821 | 0.839 |
| *65-74* | 0.797 | 0.838 | 0.810 | 0.816 | 0.804 | 0.807 | 0.820 | 0.788 | 0.823 | 0.839 |
| *75 +* | 0.756 | 0.747 | 0.752 | 0.792 | 0.752 | 0.752 | 0.780 | 0.714 | 0.788 | 0.770 |
| **Female** | 1996 | 2003 | 2004 | 2005 | 2006 | 2008 | 2010 | 2011 | 2012 | 2014 |
| *16-24* | 0.897 | 0.933 | 0.939 | 0.932 | 0.936 | 0.930 | 0.935 | 0.900 | 0.922 | 0.905 |
| *25-34* | 0.898 | 0.920 | 0.916 | 0.916 | 0.924 | 0.909 | 0.902 | 0.898 | 0.923 | 0.905 |
| *35-44* | 0.871 | 0.892 | 0.884 | 0.892 | 0.897 | 0.897 | 0.877 | 0.851 | 0.882 | 0.887 |
| *45-54* | 0.843 | 0.849 | 0.844 | 0.848 | 0.846 | 0.839 | 0.839 | 0.810 | 0.845 | 0.864 |
| *55-64* | 0.780 | 0.815 | 0.790 | 0.805 | 0.812 | 0.808 | 0.808 | 0.768 | 0.798 | 0.798 |
| *65-74* | 0.763 | 0.779 | 0.772 | 0.797 | 0.780 | 0.755 | 0.791 | 0.760 | 0.790 | 0.806 |
| *75 +* | 0.706 | 0.702 | 0.710 | 0.709 | 0.704 | 0.689 | 0.696 | 0.678 | 0.709 | 0.722 |

* Based on the UK 3L value set. Reference: Dolan P. Modeling valuations for EuroQol health states. *Med Care* 1997 Nov;35(11):1095-108.
